# A novel homodimer laccase from *Cerrena unicolor* BBP6: Purification, characterization, and potential in dye decolorization and denim bleaching

**DOI:** 10.1371/journal.pone.0202440

**Published:** 2018-08-23

**Authors:** Ji Zhang, Lei Sun, Hao Zhang, Shufang Wang, Xiaoyu Zhang, Anli Geng

**Affiliations:** 1 Key Laboratory of Molecular Biophysics of MOE, College of Life Science and Technology, Huazhong University of Science and Technology, Wuhan, China; 2 Key Lab of Green Chemical Technology & High Efficient Energy Saving of Hebei Province, Hebei University of Technology, Tianjin, China; 3 School of Life Sciences and Chemical Technology, Ngee Ann Polytechnic, Singapore, Singapore; La Trobe University, AUSTRALIA

## Abstract

The white-rot fungus *Cerrena unicolor* BBP6 produced up to 243.4 U mL^-1^ laccase. A novel laccase isoform LacA was purified; LacA is a homodimer with an apparent molecular mass of 55 kDa and an isoelectric point of 4.7. Its optimal pH was 2.5, 4.0, and 5.5 when 2, 2’-Azinobis-(3-ethylbenzthiazoline-6-sulphonate) (ABTS), guaiacol, and 2, 6-dimethoxyphenol (2, 6-DMP) were used as the substrates, respectively. The optimal temperature was 60°C for ABTS and 80°C for both guaiacol and 2, 6-DMP. LacA retained 82–92% activity when pH was greater than 4 and 42%-92% activity at or below 50°C. LacA was completely inhibited by 0.1 mM L-cysteine, 1 mM Dithiothreitol, and 10 mM metal ions, Ca^2+^, Mg^2+^ and Co^2+^. LacA had good affinity for ABTS, with a *K*_m_ of 49.1 μM and a *k*_cat_ of 3078.9 s^-1^. It decolorized synthetic dyes at 32.3–87.1%. In the presence of 1-hydroxybenzotriazole (HBT), LacA decolorized recalcitrant dyes such as Safranine (97.1%), Methylene Blue (98.9%), Azure Blue (96.6%) and simulated textile effluent (84.6%). With supplemented manganese peroxidase (MnP), Mn^2+^ and HBT, the purified LacA and BBP6 fermentation broth showed great potential in denim bleaching, with an up to 5-fold increase in reflectance values.

## Introduction

White-rot fungi has an exceptional ability to produce extracellular oxidative enzymes, which is involved in lignin degradation. These enzymes, such as lignin peroxidase (LiP), manganese peroxidase (MnP) and laccase, are able to degrade a wide range of xenobiotic compounds, including textile dyes, due to low substrate specificity [1].

Laccase (benzenediol: oxygen oxidoreductase, EC 1.10.3.2), a copper-containing polyphenol oxidase belonging to the multi-copper oxidase family, was first found in the Japanese lacquer tree, *Rhus vernicifera* [2]. It has subsequently been detected in plants, lichens, insects, bacteria and fungi. However, it is primarily produced by white-rot fungi for lignin degradation [3]. Laccases can catalyze one-electron oxidation of a wide variety of phenolic and non-phenolic substrates, such as phenol or its derivatives and aromatic amines, coupled to the transfer of four electrons to molecular oxygen to form a corresponding radical, with water as a byproduct [4, 5]. In the presence of synthetic or natural compounds that act as redox mediators, such as 1-hydroxybenzotrizole (HBT), syringaldehyde, acetosyringone and vanillin, the spectrum of laccase oxidizable substrates can be expanded considerably toward nonphenolic and more recalcitrant pollutants [6–8]. Due to their interesting catalytic properties and broad substrate specificities, laccases have many actual and potential applications in a variety of industrial fields, including paper-pulp/textile bleaching, food, pharmaceutical and cosmetic industries, bioremediation and biosynthesis [3, 9–11].

Synthetic dyes are broadly used in textile dyeing and other industrial applications. A total annual production of more than 100,000 dyes, with at least 10–15% of used dyestuff, is discharged as industrial effluents and may cause substantial ecological damage [12]. Several of these dyes are very stable and recalcitrant and cannot be completely removed from effluents by conventional physical/chemical processes. Additionally, a large amount of sludge is generated after treatment and may cause secondary pollution problems [13]. To overcome this, ligninolytic enzymes from white-rot fungi, especially laccases that can degrade synthetic dyes, are being studied for their potential application in textile effluent treatments [14–16]. In the textile industry, denim blue jeans are primarily treated with sodium hypochlorite for denim bleaching. However, this process is environmentally unfriendly, not only because chlorite itself is harmful but also because the subsequent neutralization step generates large amounts of salts, leading to disposal and pollution problems [17]. The use of a fungal laccase has been intensively evaluated for denim bleaching due to its eco-friendliness [17, 18]. Enzyme companies such as Novozyme (Denmark), Prozyme^®^ LAC (China) and Hypozyme (USA) have launched laccase-based products for denim bleaching [17]. Therefore, the use of laccases in the textile industry is rapidly growing especially as applied to textile dye decolorization and denim bleaching [9, 15, 17–18].

*Cerrena unicolor* is a white-rot fungus that commonly occurs on hardwoods [19]. It aggressively attacks living trees and causes extensive white rot. *C*. *unicolor* was previously reported to be an excellent producer of laccase [19] and an ability for dye decolorization by laccases produced by *C*. *unicolor* was reported [20–22]. This study reports the production, purification, and characterization of a novel laccase from a new white-rot fungus strain *C*. *unicolor* BBP6 and the application of its laccase in decolorization of recalcitrant dyes and denim bleaching.

## Materials and methods

### Strain and culture conditions

Strain BBP6 was isolated from a Singapore rain forest based on its superior capability for Azure B decolorization. It was denoted as a *C*. *unicolor* BBP6 according to 18S rDNA sequencing and phylogenetic tree analysis [23].

A stock culture of strain BBP6 was maintained on potato dextrose agar (PDA) (Difco, Franklin Lakes, New Jersey, USA) plates at 4°C with periodic transfer. Mycelial plugs from the stock culture were transferred to fresh potato dextrose agar (PDA) plates and incubated at 28°C for 4 days. A seed culture of strain BBP6 was performed in 250-mL Erlenmeyer flasks containing 100-mL potato dextrose broth (PDB) medium. Flasks were inoculated with ten 8-mm agar plugs with well-grown mycelium from the peripheral region of a 4-day-old PDA plate and were incubated for 5 days at 150 rpm and 28°C [23]. Ten milliliter mycelium homogenate was transferred in 100 mL fermentation medium containing PDB (24 g L^-1^), wheat bran (10 g L^-1^), and tryptone (20 g L^-1^) in 250-mL Erlenmeyer flasks and was incubated at 28°C and 150 rpm. After a 3-day incubation, 0.5 mM Cu^2+^, 0.5 mM Mn^2+^ and 100 mg L^-1^ catechol were added to the culture. Laccase and MnP activities were measured daily. Crude enzyme was harvested on day 6 and day 11, respectively, for MnP and laccase purification. The crude enzyme mixture was harvested on day 9, and the strain mycelium was removed by filtration through Whatman No. 4 filter paper followed by a 0.22-μm polyethersulfone (PES) membrane. The fermentation broth (FB) supernatant was directly used for dye decolorization and denim bleaching.

### Enzyme activity assay and protein content measurement

The activities of the laccase and manganese peroxidase (MnP) were determined according to earlier reports, with slight modification [14, 24]. A mixture of 100 μL diluted crude or purified laccase and 900 μL 1 mM ABTS solution in 100 mM acetate buffer (pH 4.0) was used to determine laccase activity. The oxidation of ABTS was monitored spectrophotometrically (UVmini 1240, Shimadzu, Japan) by measuring the increase in absorbance at 420 nm (ɛ420 = 36,000 M^-1^ cm^-1^) at room temperature. MnP activity was measured by monitoring the formation of Mn^3+^-malonate-complexes at 270 nm (ɛ270 = 11,590 M^-1^ cm^-1^) in a 1-mL reaction mixture containing 40 mM malonate buffer (pH 4.8), 1 mM MnSO_4_ and 0.1 mM H_2_O_2_ at room temperature [23]. One unit of enzyme activity (U) was defined as the amount of enzyme required to produce 1 μmol oxidized substrate per min. Extracellular protein concentration in the FB supernatant was determined by the Bradford method using bovine serum albumin (BSA) as the standard.

### Protein purification and size exclusion chromatography

The pH and ionic strength of crude BBP6 FB supernatant was adjusted to be consistent with that in the 20 mM bis-Trispropane buffer (pH 7.0). Ten milliliter supernatant samples were applied to a HiPrep DEAE FF column (1.6×10 cm; GE healthcare, UK) pre-equilibrated with 20 mM bis-Trispropane buffer at pH 7.0. Unbound proteins were washed out using the same buffer, and the adsorbed proteins were eluted with 160-mL bis-Trispropane buffer (20 mM, pH 7.0) in a linear gradient of 0–0.8 M NaCl at a flow rate of 2 mL min^−1^. The wash and elution fractions were both assayed for laccase activity, as described previously. Trace laccase activity was detected in wash fractions and elution fractions (4.5 mL) with the highest laccase activity were pooled, concentrated and dissolved in ddH_2_O by ultrafiltration using a VIVASPIN TURBO 15 concentrator (30 kDa cutoff; Sartorius, Germany). The resulting 1 mL samples were stored at -20°C for further use.

Diluted purified laccase of 100 μL was loaded on a Superdex 200 Increase 10/300 GL column (1.0×30 cm; GE healthcare, UK) pre-balanced with 10 mM phosphate buffer-140 mM NaCl (pH 7.4) and eluted with 25 mL same buffer at flow rate of 0.5 mL min^-1^. Proteins were detected by UPC-900 detector at 280 nm.

All on-column procedures were performed using an ÄKTA purifier UPC 100 system (GE healthcare, UK) at room temperature.

### Electrophoresis of *Cerrena unicolor* BBP6 laccase (LacA)

Sodium dodecyl sulfate-polyacrylamide gel electrophoresis (SDS-PAGE) and PAGE under non-denaturing conditions (native-PAGE) was conducted with a 10% Mini-PROTEAN TGX precast gel using Precision Plus Protein Unstained Standards (Bio-Rad, California, USA) in a Mini Protein Tetra system (Bio-Rad, California, USA) according to the manufacturer’s instructions. SDS-PAGE protein bands were visualized using Coomassie brilliant blue staining, whereas the native-PAGE enzyme band was visualized by incubating the gel in 1 mM guaiacol in 100 mM sodium acetate buffer (pH 4.0) at room temperature for 20 min. The isoelectric point (pI) of laccase was determined using the XCell SureLock Mini-Cell electrophoresis system (Thermo Fischer Scientific, Massachusetts, USA) with Novex pH 3–10 IEF Protein Gels (Thermo Fisher Scientific, USA) and the IEF Standards with a pI 4.45–9.6 (BioRad, Hercules, USA) according to manufacturer’s instructions.

### Effects of pH, temperature on LacA activity and stability

The optimal pH for LacA activity was evaluated at varying pH values in 100 mM citrate buffer (pH 2.0–3.0), 100 mM acetate buffer (pH 3.5–5.5) and 100 mM phosphate buffer (pH 6.0–7.0) with ABTS, 2, 6-DMP and guaiacol as the substrates at room temperature. To determine the pH stability, 100 μL purified LacA was mixed with 900 μL buffers at various pH values, i.e., 100 mM citrate buffer (pH 2.0–3.0), 100 mM acetate buffer (pH 4.0–5.0) and 100 mM phosphate buffer (pH 6.0–9.0), and was incubated at 4°C for 12 hours. Afterwards, 100 μL each mixture was used to measure the residual laccase activity at pH 4 and room temperature when ABTS was used as the substrate. All assays were conducted in duplicate.

The optimal temperature for LacA activity was determined by performing an enzymatic activity assay from 20–100°C with a 10°C increment using ABTS, 2, 6-DMP and guaiacol as the substrates at their respective optimal pH values. The thermo-stability of LacA at various temperatures was investigated by incubating the solution containing 100 μL purified LacA and 900 μL phosphate buffer (100 mM, pH 7.0) at the specified temperatures between 4°C and 70°C for 12 hours, and 100 μL each mixture was used to measure the residual laccase activity at pH 4 and room temperature when ABTS was used as the substrate. All assays were conducted in triplicate.

### Effects of metal ions and inhibitors on LacA activity and stability

The effects of metal ions such as Cu^2+^, Mn^2+^, Li^+^, Ca^2+^, Mg^2+^, Co^2+^, K^+^ and Zn^2+^, as well as potential laccase inhibitors such as sodium azide (NaN_3_), sodium bromide (NaBr), L-cysteine, ethylenediaminetetraacetic acid (EDTA), sodium dodecyl sulfate (SDS), kojic acid, dithiothreitol (DTT) and dimethyl sulfoxide (DMSO) on LacA activity were studied in 100 mM acetate buffer (pH 4.0) at room temperature with 1 mM ABTS as the substrate. All metal ions were premixed with the assay mixture to a final concentration of 10 mM and 100 mM. Inhibitors were premixed with the assay mixture to attain a final concentration of 0.1 mM to 10 mM. To determine the stability of LacA in the presence of various metal ions, the purified LacA was incubated in 10 mM of each individual metal ion at 4°C for 24 hours, and the residual laccase activity was measured at pH 4 and room temperature when ABTS was used as the substrate. All measurements were conducted at least in duplicate.

### Substrate specificity of LacA

The substrate specificity of LacA was determined by measuring the laccase activity using 5–100 μM ABTS, 0.5–5 mM guaiacol, and 0.5–10 mM 2,6-DMP as the substrates at their corresponding optimal pH and temperature. The kinetic parameters of the Michaelis-Menten equation were calculated according to Lineweaver-Burk plots. Kinetic studies were performed in triplicate.

### Screening of redox mediators for synthetic dye decolorization

Dye decolorization was performed in 10-mL reaction mixtures composed of 100 mM acetate buffer (pH 4.0), 500 U L^-1^ purified or crude laccase, 2 mM redox mediators, such as vanillin, hydroxybenzotriazole (HBT), 3,5-diaminobenzoic acid and syringaldehyde and 50 mg L^-1^ each individual dye [16, 25]. Reaction mixtures without laccase (with or without redox mediators) were used as controls for each dye. Dye decolorization was monitored by measuring the absorbance at 429 nm for Phenol Red, 552 nm for Brilliant Blue R (BBR), 553 nm for Safranin, 560 nm for Congo Red, 589 nm for Crystal Violet, 592 nm for Bromophenol Blue, 612 nm for Xylene Cyanol, 615 nm for Remazol Brilliant Blue R (RBBR), 623 nm for Fast Green FCF, 644 nm for Azure B and 664 nm for Methylene Blue. The optimal wavelength for each individual dye was determined in 100 mM acetate buffer at 380 to 800 nm. Reaction mixtures were incubated at room temperature for 24 hours, and the absorbance at the maximum wavelength of each dye was recorded periodically. Dye decolorization was calculated according to the following formula: Decolorization (%) = [(Ai–At)/Ai] × 100, where Ai is the initial absorbance of the dye, and At is the absorbance at the time of measurement. All experiments were conducted in duplicate.

### Dye decolorization in simulated textile effluent (STE)

STE was prepared according to a previous report [26] with 50 mg L^-1^ each the above dye, 30 g L^-1^ NaCl, 5 g L^-1^ Na_2_CO_3_ and 0.4875 g L^-1^ NaOH, with a final pH of 4.5. The dye decolorization reaction mixture contained 2 mM HBT, 10 U mL^-1^ laccase and 50% or 100% (v/v) STE in a total volume of 2 mL. The reaction mixtures were incubated at 150 rpm and room temperature for 72 hours, and dye decolorization in STE was measured spectrophotometrically at 380 to 700 nm. Reaction mixtures without laccase were used as controls.

### Co-operation of LacA and MnP in denim bleaching

A series of denim bleaching solutions (DB Solutions) at pH 4.0 were prepared to study the cooperation between LacA and MnP in denim bleaching (Table 1). MnP was purified according to our previous report [23]. Denims A-G were purchased in a retail shop in Singapore and used for the experiments. Denim pieces (4 × 4 cm) were individually immersed in 30 mL of each DB solution and incubated at 30°C and 100 rpm for 24 hours. After treatment, denim samples were rinsed with running tap water and dried at 60°C in an oven. Color reduction was quantified using a CM-600D reflectance spectrophotometer (Konica Minolta Inc., Japan). All experiments were conducted in duplicate.

**Table 1:** Denim bleaching solutions with laccase and MnP from *C*. *unicolor* BBP6.

DB Solution	100 mM acetate buffer	2 mM HBT	10 U mL^-1^ LacA	40 mM malonate buffer	1 mM Mn^2+^	100 U L^-1^ MnP
I	✓	✓	x	x	x	x
II	✓	✓	✓	x	x	x
III	x	✓	✓	✓	✓	x
IV	x	✓	✓	✓	✓	✓

For comparison, BBP6 FB supernatant obtained as described previously was directly used for denim bleaching. In this case, the DB solution contained FB supernatant (10 U mL^-1^ laccase and 71.4 U L^-1^ MnP), 1 mM Mn^2+^ and 2 mM HBT in 40 mM malonate buffer (pH 4.0). The reference treatment (blank) was conducted under the same conditions without FB supernatant.

## Results and discussion

### Laccase production, purification, molecular mass and *pI* estimation

**Fig 1:**
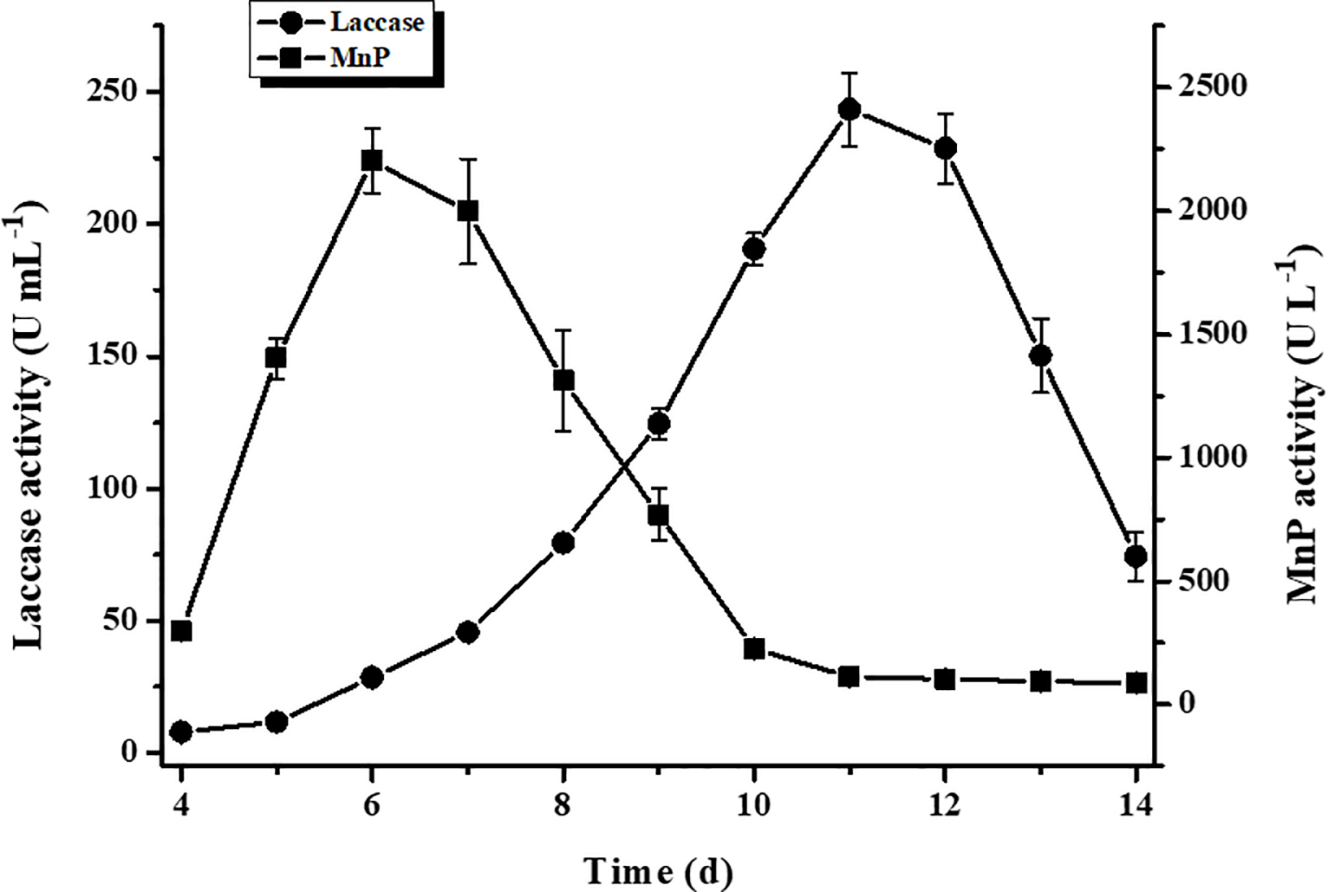
Laccase and MnP production profile for *C*. *unicolor* BBP6. Bars indicate standard deviations (± 15%) of triplicate determination.

**Fig 2:**
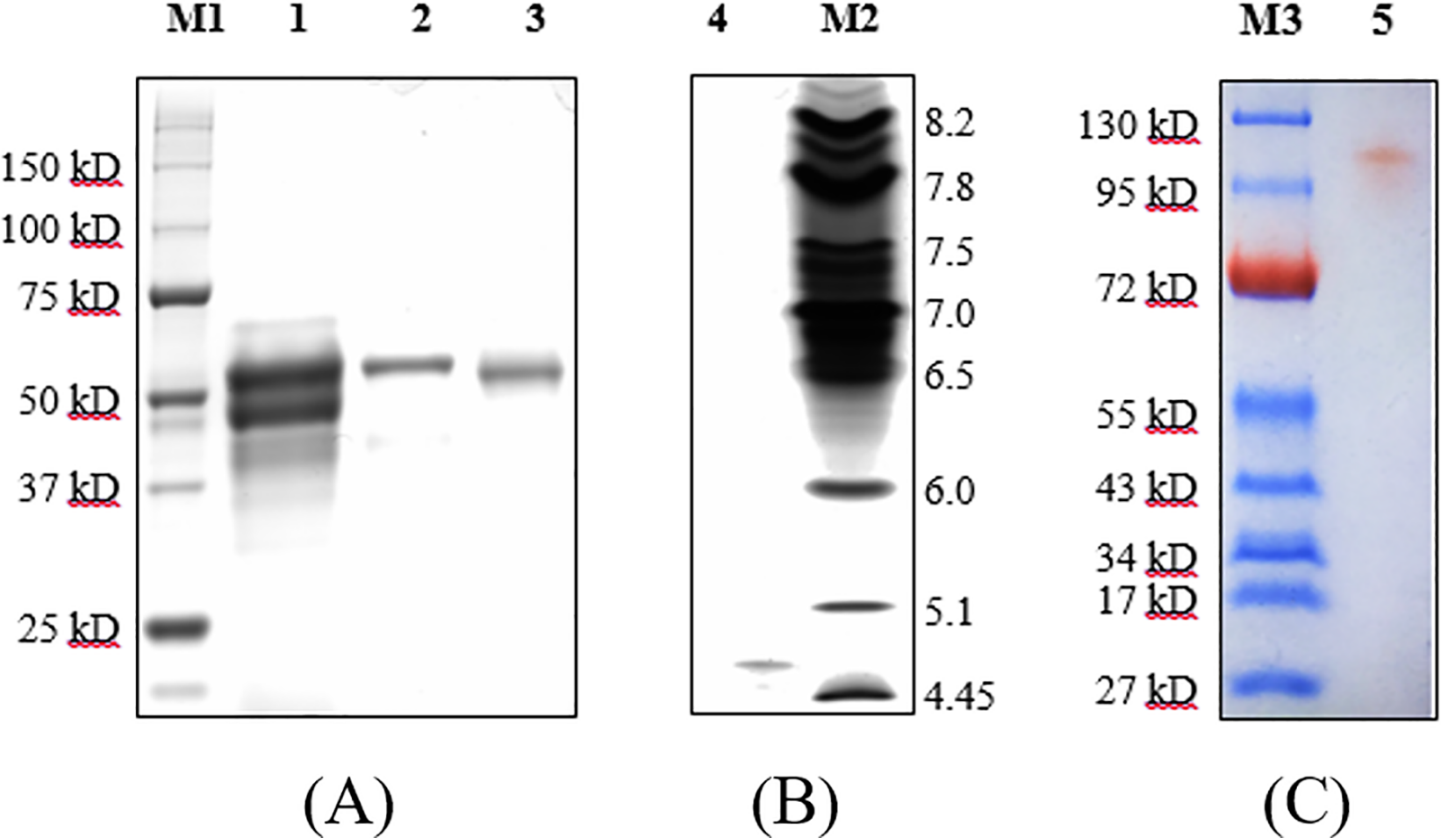
**SDS-PAGE (A), isoelectric focusing (B) and native-PAGE (C) of purified LacA.** Lane M1, Precision Plus Protein Unstained Standards (Bio-rad); lane 1, crude laccase; lane 2, purified LacA; lane 3, purified LacA after 2-mercaptoethanol treatment; lane 4, purified LacA; lane M2, IEF Standards, pI 4.45–9.6 (BioRad); lane M3, Pageruler prestained protein ladder (Thermo Fisher Scientific); lane 5, purified LacA.

Laccase and MnP production profiles are displayed in Fig 1. Laccase and MnP activity, respectively, peaked at 243.4 U mL^-1^ on day 11 and 2202.6 U L^-1^ on day 6. Crude laccase samples were harvested on day 11, were frozen and were subsequently thawed to remove long-chain polysaccharides. The specific laccase activity of crude laccase was 629.7 U mg^-1^ (Table 2). Laccase was purified to homogeneity through anion exchange chromatography followed by ultrafiltration using a 30-kDa cutoff, until a monomeric laccase designated LacA was obtained. Crude BBP6 laccase was purified 1.9-fold in 81.4% yield and with 1215.9 U mg^-1^ specific laccase activity (Table 2). These are comparable to the reported data for *Cerrena* sp. WR1 laccase (1013.5 U mg^-1^) [20] and that for *Cerrena* sp. HYB07 laccase (1155.0 U mg^-1^); the latter was obtained with similar purification fold (1.8) but less yield (73.5%) [21]. Further purification improved specific activity and purification fold for *Cerrena* sp. HYB07 laccase respectively to 1952.4 U mg^-1^ and 3.1, however, with much lower yield (39.8%) [21]. SDS-PAGE displayed two major bands for crude laccase and only a single band for purified LacA (Fig 2A), indicating its homogeneity. The above results suggest a high LacA content of crude BBP6 laccase. Zymography demonstrated an orange single band with a molecular mass of 110 kDa (Fig 2C), whereas the molecular mass of the reducing and non-reducing LacA were 53 and 55 kDa, respectively. (Fig 2A). The isoelectric point (pI) of LacA was determined to be 4.7 (Fig 2B), which is slightly higher than the pI of 3.5–4.0 reported for fungal laccases [27].

**Table 2:** Purification of extracellular laccase from *C*. *unicolor* BBP6.

Purification step	Total protein (mg)	Total activity (U)	Specific activity (U mg^-1^)	Purification fold	Yield (%)
Crude laccase	14.1 ± 0.6	8897.8 ± 363.3	629.7 ± 25.7	1	100
DEAE FF	6.0 ± 0.1	7240.0 ± 116.4	1215.9 ± 19.5	1.9	81.4%

Values are presented as the means of ± SD of triplicate.

**Fig 3:**
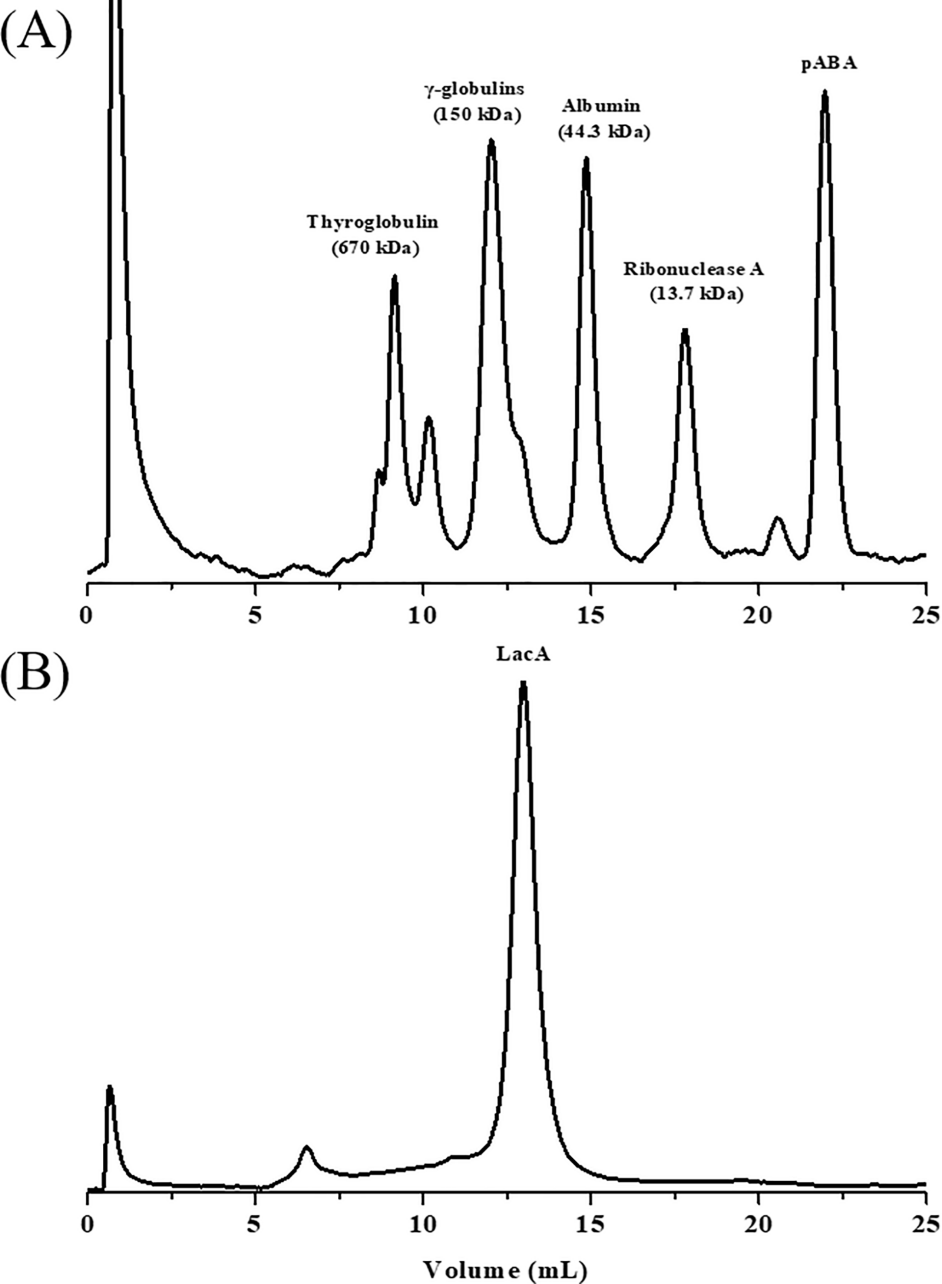
Size exclusion chromatography of native LacA.

LacA showed a single band at 55 kDa by SDS-PAGE. In addition, the molecular mass of the purified native LacA was estimated to be 108 kDa by size exclusion chromatography (Fig 3), suggesting that LacA might be a disulfide- or hydrogen-linked homodimer. Similar results were also reported for laccases from other fungal species [28–30]. The WRF strain, *Phellinus ribis*, produced a single form of laccase, and this laccase was a dimer consisting of two 76-kDa subunits [28]. The laccase from a tree legume, *Leucaena leucocephala*, was found to be a heterodimer (∼220 kDa), which contained two subunits with respective molecular masses of 100 and 120 kDa [29]. Otto and Schlosser reported a laccase from an algal strain, *Tetracystis aeria* [30]. The native enzyme (apparent molecular mass of ~220 kDa) was a hetero-oligomer with a composition of AB_2_, where A (~110 KDa) is the catalytic subunit and B_2_ (71 KDa) is a disulfide-linked homodimer. LacA is a homodimer of two subunits, with a molecular mass of 55 kDa. To date, no such results have been reported for laccases obtained from other *Cerrena* species.

### Effects of pH and temperature on LacA activity

**Fig 4:**
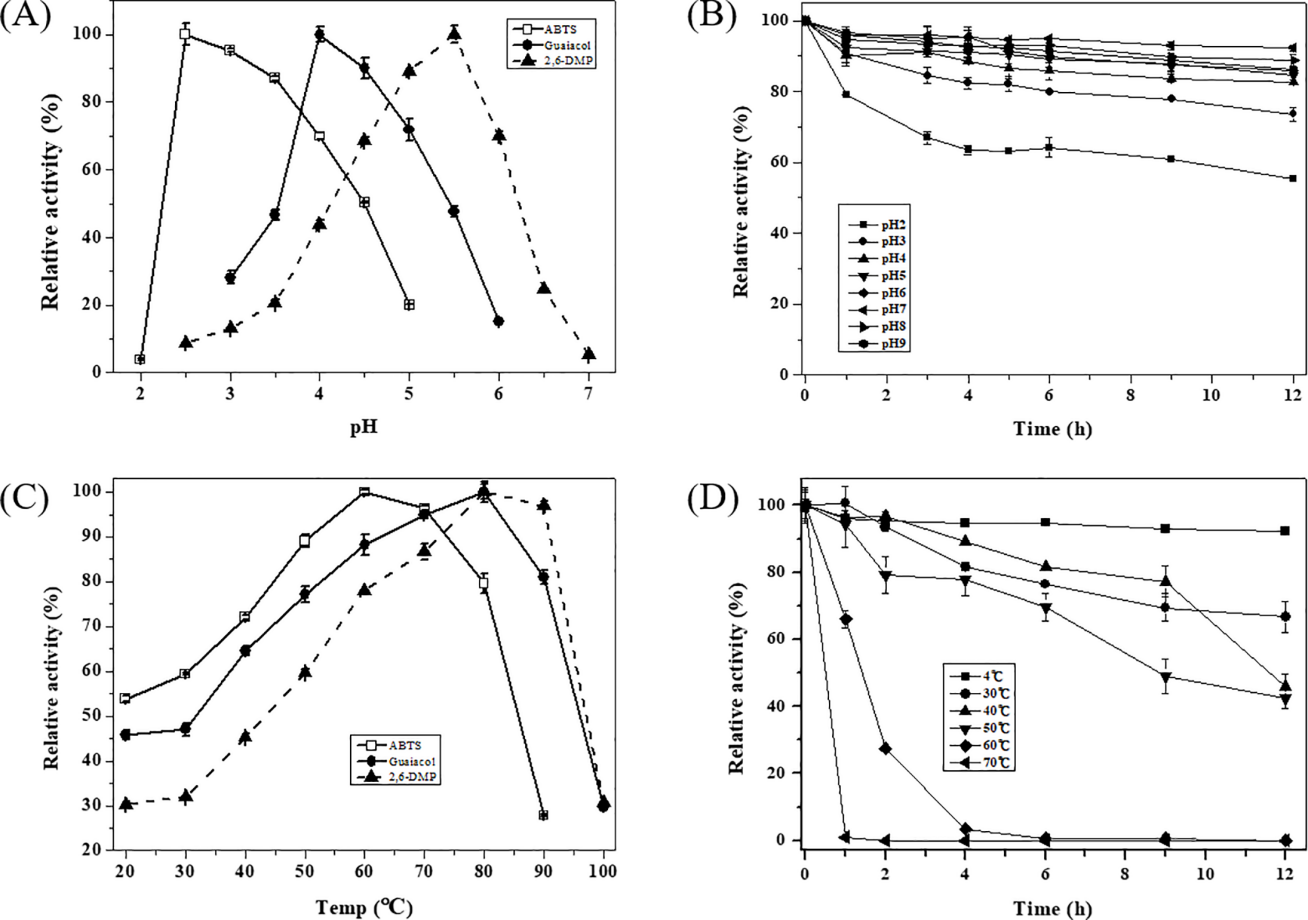
Effects of pH and temperature on activity and stability of LacA. (A) Effect of pH on LacA activity with ABTS, guaiacol and 2,6-DMP as the substrates. (B) Effect of pH on LacA stability with ABTS as substrate. (C) Effect of temperature on LacA activity with ABTS, guaiacol and 2,6-DMP as substrates. (D) Effect of temperature on LacA stability with ABTS as substrate. Bars indicate standard deviations (± 10%) of at least duplicate determination.

The optimal pH for LacA activity was 2.5 for ABTS, 4.0 for guaiacol and 5.5 for 2, 6-DMP. For pH above 6, laccase activity decreased rapidly and LacA was almost completely deactivated at pH 7 (Fig 4A). This was associated with the difference in redox potential between the reducing substrates and the type I copper in the active site of the enzyme, as well as with the hydroxide ion inhibition of type III copper at higher pH, as suggested by Xu [31]. LacA was relatively unstable at pH below 4.0. Its activity decreased to 55.2% and 73.7% original enzyme activity after a 12-h incubation at pH 2.0 and 3.0, respectively. In contrast, LacA retained more than 82% original activity when incubated at a pH of 4.0 or above (Fig 4B). Similar results of *Cerrena* sp. laccases were reported by Chen et al [20] and Yang et al [21] previously.

The optimal temperature for LacA was 60°C for ABTS and 80°C for both guaiacol and 2,6-DMP, higher than that for other *Cerrena* sp. laccases [20, 21]. LacA activity steadily grew as the temperature increased from 30°C to 60°C and decreased drastically above 80°C (Fig 4C). LacA was almost deactivated after 1-h incubation at 70°C and 4-h incubation at 60°C. However, approximately 42%-92% original activity was retained after 12-h incubation at 50°C and below (Fig 4D). It is worth noting that more than 80% original activity was retained for LacA after 2-h incubation at 50°C (Fig 4D). Whereas, laccases from *Cerrena* sp. WR1 and *Trametes pubescens* respectively were able to retain 50% and 72% original activity under the same conditions [20, 25]. This suggests that LacA had good thermos-stability.

### Effects of inhibitors and metal ions

Among all of the investigated inhibitors, L-cysteine was the strongest, as no residual activity was detected at 0.1 mM (Table 3). LacA was also completely inhibited by 1 mM NaN_3_, 1 mM DTT and 10 mM SDS. The inhibitory effect increased with increasing concentration of most inhibitors, except DMSO. The relative LacA activity decreased to 73.3%, 71.4% and 85.2% original activity in the presence of 10 mM NaBr, 10 mM EDTA and 10 mM kojic acid, respectively.

**Table 3:** Effects of inhibitors on LacA activity.

Inhibitor	Concentration (mM)	Relative activity (%)
None	-	100
NaN_3_	0.1	1.15 ± 0.04
	1	0
	10	0
NaBr	0.1	79.62 ± 4.94
	1	83.07 ± 0.52
	10	73.30 ± 2.72
L-cysteine	0.1	0
	1	0
	10	0
EDTA	0.1	82.72 ± 4.10
	1	79.34 ± 1.66
	10	71.43 ± 0.88
SDS	0.1	70.52 ± 2.87
	1	41.77 ± 1.23
	10	0
Kojic acid	0.1	99.24 ± 1.03
	1	89.31 ± 0.83
	10	85.24 ± 0.18
DTT	0.1	22.80 ± 2.00
	1	0
	10	0

Values are presented as the means of ± SD of duplicate.

The above inhibition studies proved that LacA was not sensitive to NaBr, chelator EDTA and kojic acid, but was strongly inhibited by L-cysteine, NaN_3_, SDS and DTT. It was reported that the binding of NaN_3_ to type II and type III copper sites affected internal electron transfer, thereby inhibiting laccase activity [32]. The anionic detergent SDS might cause a conformational change in LacA, leading to the inhibition of this enzyme [33]. However, Christian Johannes and Andrzej Majcherczyk] reported that the apparent inhibitory effects of L-cysteine and DTT were primarily caused by reduction of ABTS radical cation or non-enzymatic interactions with unreacted ABTS rather than by the inhibition of laccase [34].

**Fig 5:**
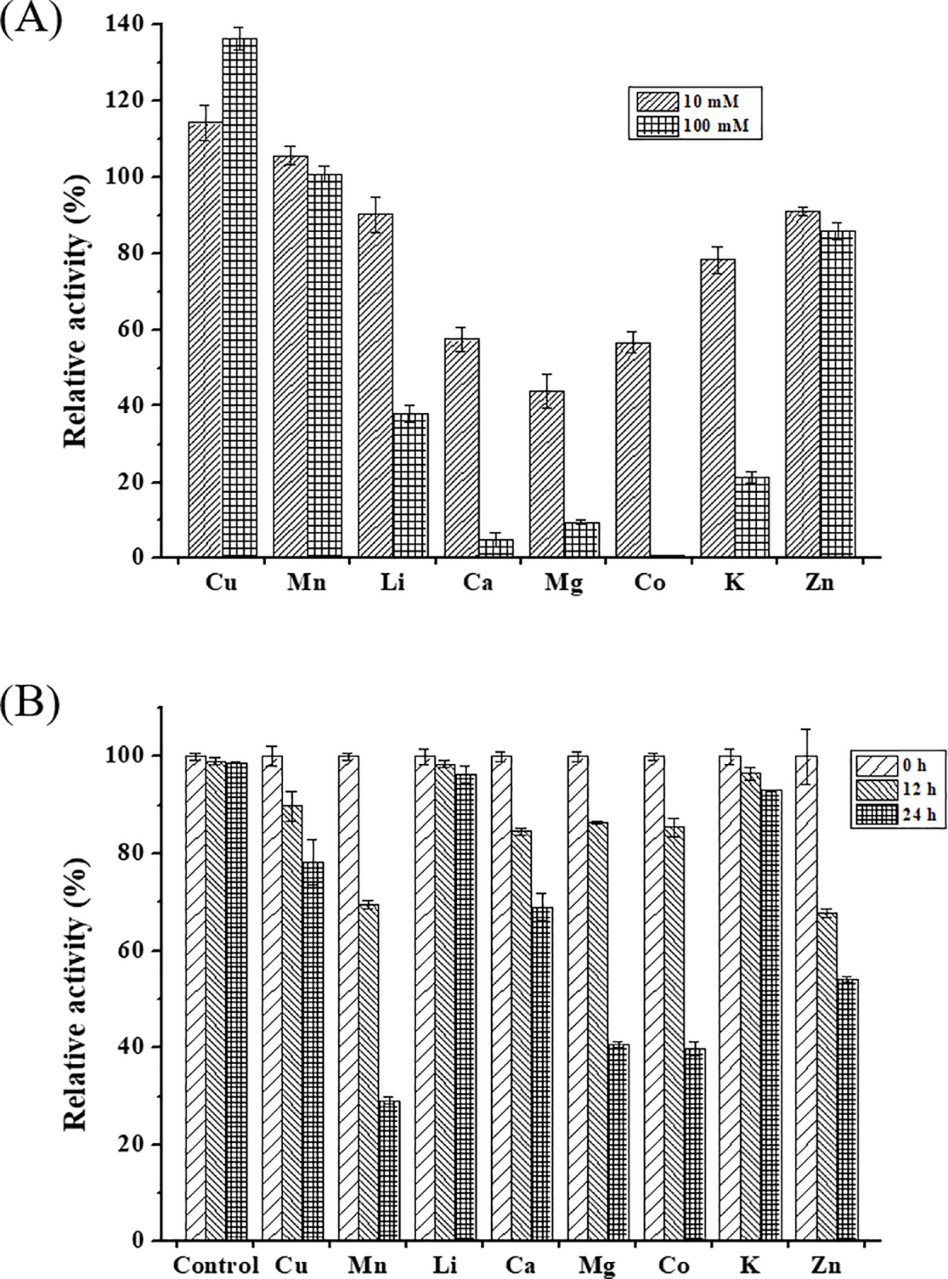
**Effects of metal ions on LacA activity (A) and LacA stability (B).** Bars indicate standard deviations (± 10%) of at least duplicate determination.

Metal ions had opposite effects on LacA activity. LacA activity was slightly enhanced by Cu^2+^ (Fig 5A), which can be attributed to the filling of type II copper binding sites with copper ions [35]. It was inhibited by Ca^2+^, Mg^2+^ and Co^2+^, with losses of approximately 42–56% original activity at 10 mM, and greater than 90% original activity loss at 100 mM. Interestingly, no significant activity loss was observed at 10 mM and 100 mM Mn^2+^ (Fig 5A).

Overall, all metal ions tested had negative effects on laccase stability (Fig 5B). LacA was relatively stable in the presence of Li^+^ and K^+^, which retained over 90% original laccase activity after 24-h incubation. Cu^2+^, Mn^2+^, Ca^2+^, Mg^2+^, Co^2+^ and Zn^2+^ reduced LacA relative activity to 29–78% (Fig 5B). Interestingly, Cu^2+^ decreased LacA stability, even though laccase is a copper-containing enzyme. This is in agreement with a previous report. Lorenzo et al. indicated that the addition of Cu^2+^ into the reaction mixture stimulated laccase activity at concentrations lower than 1 mM but significantly inhibited activity at concentrations ranging from 2–80 mM. Copper acts as enzyme co-factors simulating activity at certain levels; however, it is toxic when present in excess [36].

### Substrate specificity

ABTS presented the lowest apparent *K*_*m*_ (49.11 μM) compared to guaiacol and 2,6-DMP (Table 4), suggesting a high affinity of LacA to ABTS. In addition, the highest turnover rate (*k*_*cat*_) and catalytic efficiency (*k*_*cat*_/*K*_*m*_) were 3078.94 s^-1^ and 62.7 μM^-1^ s^-1^, respectively, when ABTS was used as the substrate. When guaiacol was used as the substrate, lower *k*_*cat*_ (149.79 S^-1^) and *k*_*cat*_/*K*_*m*_ (0.12 μM^-1^ S^-1^) values were obtained, while the lowest values were obtained for 2, 6-DMP with a *k*_*cat*_ of 11.29 s^-1^ and a *k*_*cat*_/*K*_*m*_ of 3.29×10^−3^ μM^-1^s^-1^. In agreement with a recent report [15], LacA possessed relatively high affinity and catalytic efficiency towards ABTS, followed by guaiacol and 2, 6-DMP (Table 4).

**Table 4:** Kinetic parameters of LacA under optimal conditions.

Substrate	Wavelength (nm)	ε (M^-1^ cm^-1^)	*K*_m_ (μM)	*k*_cat_ (s^-1^)	*k*_cat_/*K*_m_ (μM^-1^ S^-1^)
ABTS	420	36,000	49.1	3078.9	62.7
Guaiacol	465	12,000	1238.6	149.8	0.1
2,6-DMP	469	49,600	3430.8	11.3	3.3×10^−3^

Asymptotic significance of Lineweaver-Burk plots for all substrates (p < 0.001).

### Effects of redox mediators on synthetic dye decolorization by crude laccase

Notably, the best dye decolorization results were obtained using 1-hydroxybenzotriazole (HBT) as the redox mediator (Table 5), in good agreement with previous reports [37–38]. HBT presented more than 90% dye decolorization for Fast Green FCF, Xylene Cyanol and Bromophenol Blue, while the lowest decolorization was 74–78% for Phenol Red Safranin and Congo Red. However, syringaldehyde improved Congo Red, Crystal Violet and Bromophenol Blue decolorization to 84.2%, 83.1%, and 80.9%, respectively, close to values obtained with HBT. Vanillin enhanced Bromophenol Blue decolorization to 84.02% and 3, 5-diaminobenzoic acid improved Congo Red decolorization to 76.38%. Although the other mediators were also active, their dye decolorization efficiency was lower than that obtained by HBT (Table 5).

**Table 5:** Effects of redox mediators on synthetic dye decolorization by crude laccase in 24 h.

Dye	No mediator	HBT	Vanillin	3,5-diaminobenzoic acid	Syringaldehyde
Fast Green FCF	0.55	92.84	9.56	6.29	10.23
Congo Red	22.11	77.68	67.82	76.38	84.18
Methylene Blue	8.47	88.24	6.33	9.45	6.86
Safranine	8.19	74.44	21.09	3.72	24.32
Azure B	0.69	81.49	1.86	14.69	6.95
Crystal Violet	23.83	83.79	73.56	65.51	83.13
Phenol Red	26.32	73.99	26.79	6.85	26.17
Xylene Cyanol	81.10	92.78	54.98	75.81	82.52
Bromophenol Blue	41.37	93.70	84.02	57.63	80.91
Brilliant Blue R	25.80	80.14	37.97	9.61	58.36
Remazol Brilliant Blue R	68.46	87.70	76.05	7.46	69.11

### Dye decolorization by purified LacA

**Fig 6:**
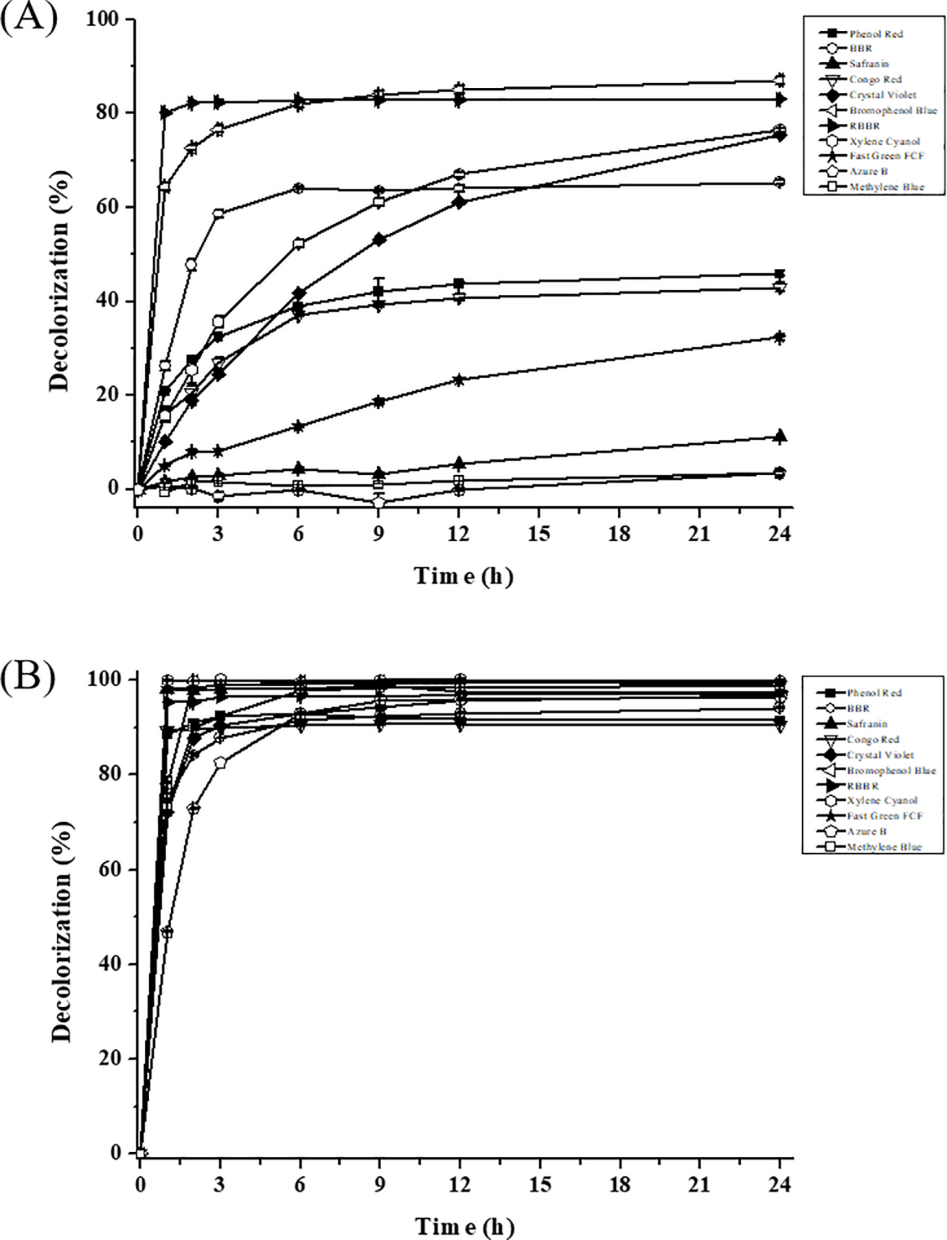
Dye decolorization with purified LacA (500 U L^-1^). (A) Without HBT. (B) With HBT. Bars indicate standard deviations (± 10%) of duplicate determination.

Decolorization capability of purified LacA on industrial and laboratory dyes was evaluated at a laccase activity of 500 U L^-1^ (Fig 6A). Among the 11 investigated dyes, eight were decolorized by more than 30% in the absence of redox mediator. RBBR, a representative anthraquinone dye, was rapidly decolorized by over 80% in 1 hour, while Bromophenol Blue decolorization reached 87.1% in 24 hours. Phenol Red, BBR, Congo Red, Crystal Violet, Xylene Cyanol and Fast Green FCF were decolorized by 32–76% in 24 hours (Fig 6A). Notably, no decolorization was observed for Safranin, Azure B and Methylene Blue.

HBT was introduced to enhance dye decolorization as the redox mediator (Fig 6B). The addition of 2 mM HBT greatly enhanced decolorization efficiency and remarkably shortened decolorization time (Fig 6B). LacA could rapidly decolorize almost all dyes, with greater than 80% decolorization in 3 hours. In addition, Bromophenol Blue, Xylene Cyanol, Fast Green FCF and Methylene Blue were almost completely decolorized. Azure B, one of the most recalcitrant heterocyclic dyes, was decolorized by 93.0% and 96.6%, respectively, in 6 and 24 hours. This was the first report on Azure B decolorization by a purified laccase from a *Cerrena* species.

Azure B is a selective substrate for the detection of lignin peroxidase activity, and only a few laccases can oxidize it. High redox-potential laccases purified from *Pycnoporus* sp. [6, 37, 39] and *Polyporus pinsitus* (*Trametes villosa*) [40] were able to decolorize Azure B (Table 6). The highest Azure B decolorization (90% in 1 hour) was obtained by laccase purified from *T*. *trogii* BAFC 463 in the presence of 0.5 mM HBT, with 6,500 U L^-1^ laccase and 5.5 mg L^-1^ Azure B [38]. In our study, greater than 82% Azure B decolorization was obtained using a much lower laccase concentration (500 U L^-1^) and higher Azure B concentration (50 mg L^-1^) (Table 6). These results suggest that LacA has great potential in recalcitrant dye decolorization.

**Table 6:** Comparison of Azure B decolorization ability.

Species	Laccase activity (U L^-1^)	Azure B (mg L^-1^)	Mediator	Decolorization (%)	Reference
*Cerrena unicolor* BBP6	500	50	2 mM HBT	82.6 (3 h) 93.0 (6 h) 96.6 (24 h)	This study
*Trametes trogii* BAFC 463	6,500	~5.5	0.5 mM HBT	90 (1 h)	[38]
*Pycnoporus coccineus* BRFM 938	4,000	40	No	11 (52 h)	[37]
*Pycnoporus sanguineus* BRFM 902	4,000	40	0.9 mM HBT	11 (52 h)	
*Pycnoporus sanguineus* BRFM 66				21 (52 h)	
*Pycnoporus cinnabarinus* SS3	100	~7.6	0.25 mM ρ-coumaric acid	57.6 (2 h)	[6]
			0.1 mM acetosyringone	40 (2 h)	[39]
			0.1 mM acetosyringone	40 (2 h)	
*Polyporus pinisitus* (*Trametes villosa*)	2,400	200	2 mM HBT	73 (16 h)	[40]

**Fig 7:**
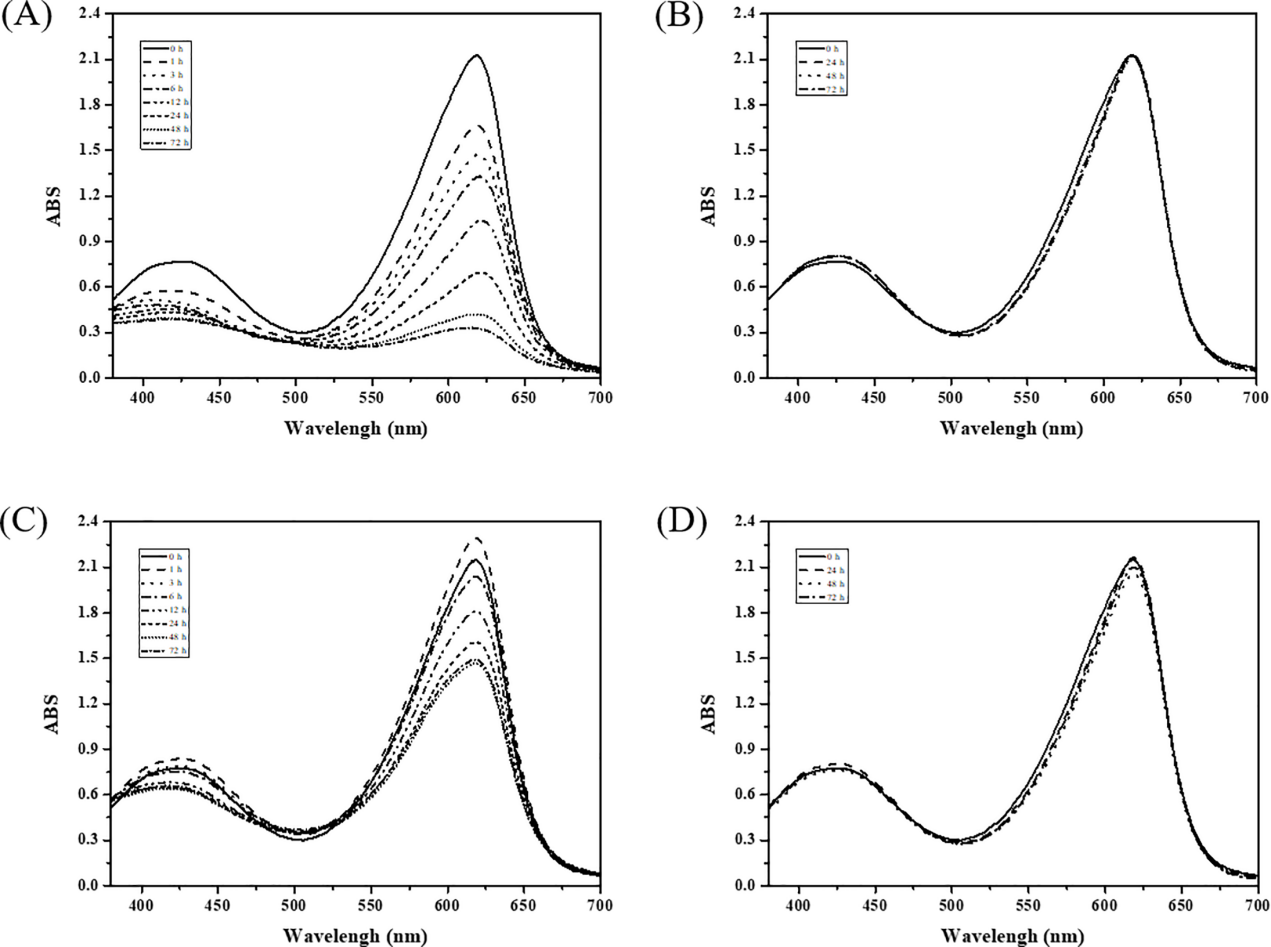
Dye decolorization in simulated textile effluent (STE) by purified laccase in presence of 2 mM HBT. (A) 50% STE with laccase; (B) 50% STE without laccase; (C) 100% STE with laccase; (D) 100% STE without laccase. (p < 0.001).

Simulated textile effluent (STE) somewhat mimics industrial textile effluent [23]. STE samples containing a mixture of 11 synthetic dyes were decolorized by purified LacA (Fig 7). As seen, STE samples had the highest adsorption at 619 nm. When the total dye concentration was 225 mg L^-1^ (50% STE), approximately 85% dye decolorization was obtained at 72 h (Fig 7A). This decreased to 30% when the total dye concentration reached 550 mg L^-1^ (100% STE) (Fig 7C). No dye decolorization was observed in STE samples without laccase (Fig 7B and 7D). Dye mixture decolorization in artificial textile effluent was previously investigated using WRF laccases. The immobilized laccase from white-rot fungus *Pleurotus florida* NCIM 1243 was able to decolorize 85% of simulated dye effluent containing 0.5 mM of a mixture of five reactive dyes [41]. In the presence of HBT, the purified LacA was able to decolorize approximately 85% of the dye mixture in STE, with a total dye concentration of 225 mg L^-1^, suggesting that the LacA-HBT system has potential in textile effluent decolorization.

### Denim bleaching by purified and crude laccase

**Fig 8:**
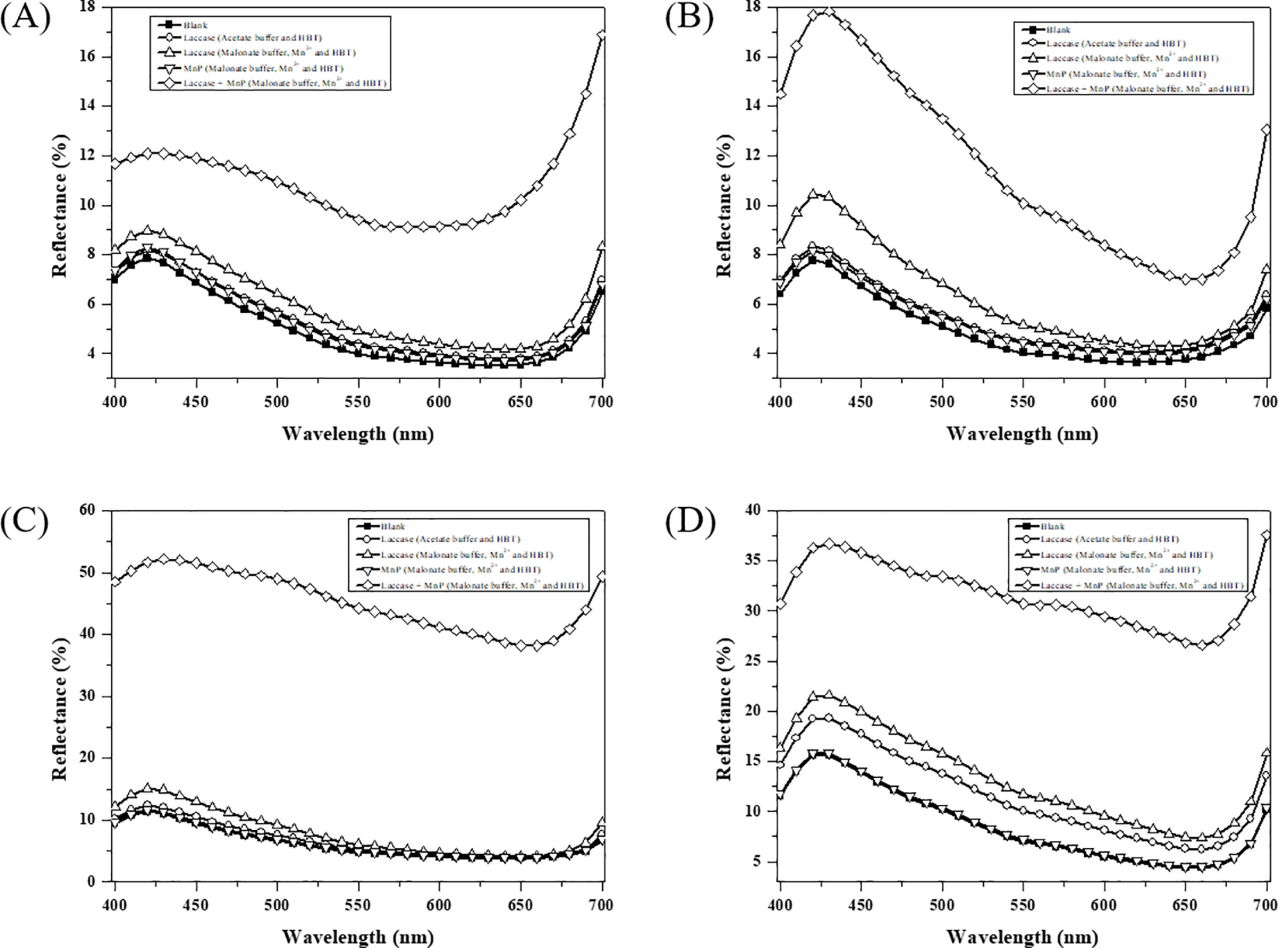
**Reflectance spectrums of denims A-D (A-D) treated with purified LacA and MnP.** Reaction solutions: (I) 100 mM acetate buffer (pH 4.0) and 2 mM HBT; (II) 10 U mL^-1^ LacA, 100 mM acetate buffer (pH 4.0) and 2 mM HBT; (III) 10 U mL^-1^ LacA, 40 mM malonate buffer (pH 4.0), 1 mM Mn^2+^ and 2 mM HBT; (IV) 10 U mL^-1^ LacA, 100 U L^-1^ MnP, 40 mM malonate buffer (pH 4.0), 1 mM Mn^2+^ and 2 mM HBT. (p < 0.001).

Color reduction was quantified as the reflectance increased, as measured using a reflectance spectrophotometer at 400–700 nm (Fig 8). When LacA in acetate buffer was used (DB Solution II in Table 1), denim D presented an approximately 3% reflectance increase compared to the blank (DB Solution I), whereas the other rest denims displayed almost no reflectance differences. This indicates that the LacA-HBT system alone is not effective for denim bleaching. Schlosser and Höfer [24] reported that in the presence of Mn^2+^ and chelator, laccase was able to produce Mn^3+^-chelate and generate H_2_O_2_, and thereby initiated MnP reactions.

Accordingly, denim bleaching was performed in malonate buffer with and without MnP. Interestingly, when LacA was in malonate buffer with Mn^2+^ and HBT (DB Solution III) there was an approximately 1–2% reflectance increase for denims A-C and an approximately 4–8% reflectance increase was obtained for denim D. Compared to treatment with LacA in acetate buffer, the improved color reduction might be due to the Mn^3+^-chelate generated by LacA. However, when MnP was added to the above LacA solution (DB Solution IV), a significant enhancement in denim bleaching was observed. The reflectance increase of 4–9% and 3–10% was obtained for denim A and B, respectively. Approximately 40% and 23% reflectance increases were respectively obtained for denim C and D. MnP in malonate buffer with 1 mM Mn^2+^ and 0.1 mM H_2_O_2_ was effective for denim bleaching without mediators [23]. MnP alone, without H_2_O_2_, in malonate buffer with Mn^2+^ and HBT presented almost no denim bleaching effects (Fig 8). The above results suggest that LacA not only contributes to denim bleaching but also plays a role in H_2_O_2_ provision. The latter subsequently initiates the MnP reaction.

**Fig 9:**
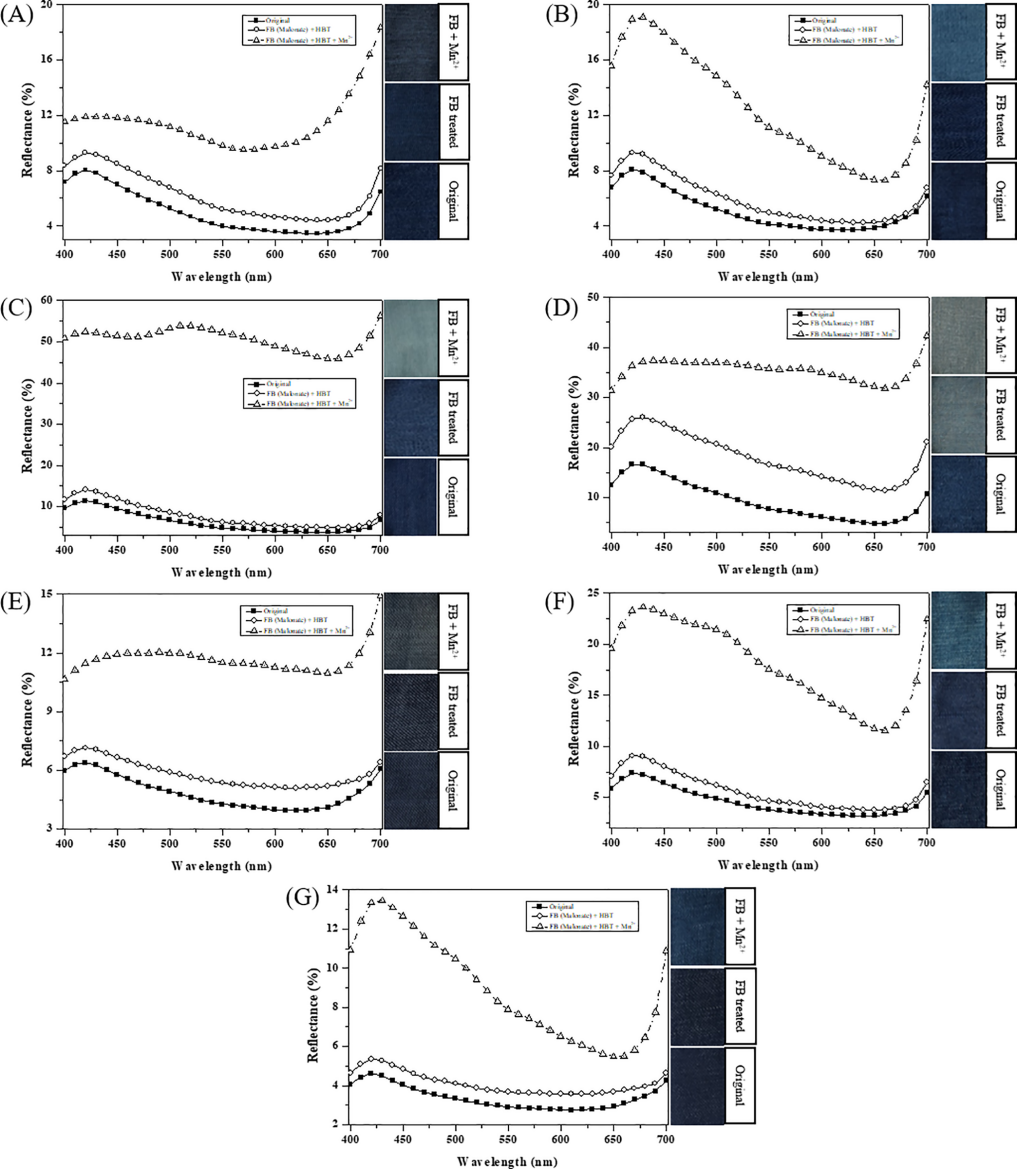
**Reflectance spectrums and pictures of denims A-G (A-G) treated with fermentation broth derived from *C*. *unicolor* BBP6.** Reaction solution contained 40 mM malonate buffer (pH 4.0), 1 mM Mn^2+^, 2 mM HBT, 10 U mL^-1^ laccase and 71.4 U L^-1^ MnP. (p < 0.001).

Based on the above evidences, the BBP6 strain FB may be a good alternative to purified LacA for denim bleaching, as it contains both laccase and MnP (Fig 1). Therefore, denim-bleaching experiments were conducted using the supernatant of BBP6 FB harvested on day 9, which contained 10 U mL^-1^ laccase and 71.4 U L^-1^ MnP. Additional denims (E-G) were also tested (Fig 9). Denim bleaching efficiency was low (approximately 1–2% reflectance increase) without Mn^2+^ and was greatly enhanced with the addition of Mn^2+^ and chelator. Denim A exhibited a reflectance increase (4–6%) at 400 to 600 nm lower than that obtained at 650–700 nm (8–11%) (Fig 9A). However, denim B, F and G respectively presented 9–11%, 15–16% and 7–8% reflectance increases at 400–500 nm higher than those obtained at 600–700 nm (3–5%, 8–11% and 2–4%) (Fig 9B, 9F and 9G). Denim E presented a relatively low bleaching effect (approximately 5–7% reflectance increase) over the full wavelength range (Fig 9E). In contrast, remarkable denim bleaching effects were observed for denim C and D (Fig 9C and 9D). The reflectance increase for denim D was 19–30% but was over 40% (41–46%) for denim C over the entire wavelength range. These corresponded to 2- to 3- and 4- to 5-fold increases compared to their respective initial reflectance. As other laccase isoforms, enzymes and small molecules existing in crude BBP6 FB might also played some roles in denim bleaching [42, 43], bleaching effects were slightly better than those obtained using purified LacA with MnP (DB Solution IV). This was superior to an earlier report where reflectance increases of 10–28% were reported when the fungal crude extract (FCE) derived from *Pycnoporus sanguineus* with violuric acid (VA) was used [18].

The above results demonstrate that *Cerrena unicolor* BBP6 FB is an effective agent and a potential alternative to the commercial enzyme formulations currently used for denim bleaching. These significant denim bleaching effects could be largely attributed to the synergistic interaction between LacA and MnP.

## Conclusion

A *Cerrena unicolor* strain BBP6 was isolated from the Singapore rain forest and produced 243.4 U mL^-1^ laccase on day 11 and 2202.6 U L^-1^ MnP on day 6. A major laccase isoform (LacA) was purified. Biochemical characterization revealed that LacA had high specific activity, high substrate specificity to ABTS, good thermo- and pH-stability, potential tolerance to typical inhibitors and metal ions, and promising dye decolorization ability. Both crude BBP6 laccase and purified LacA displayed excellent dye decolorization capabilities, as demonstrated by synthetic dye decolorization and denim bleaching, especially in the presence of the redox mediator, HBT, and Mn^2+^. This remarkable dye decolorization and denim bleaching ability suggests that *Cerrena unicolor* BBP6 laccase or BBP6 crude fermentation broth has great application potential for the textile industries.
